# Working memory and inhibitory control deficits in children with ADHD: an experimental evaluation of competing model predictions

**DOI:** 10.3389/fpsyt.2024.1277583

**Published:** 2024-05-08

**Authors:** Michael J. Kofler, Nicole B. Groves, Elizabeth S. M. Chan, Carolyn L. Marsh, Alissa M. Cole, Fatou Gaye, Enrique Cibrian, Miho O. Tatsuki, Leah J. Singh

**Affiliations:** ^1^ Department of Psychology, Florida State University, Tallahassee, FL, United States; ^2^ Department of Psychiatry, Seattle Children’s Hospital, Seattle, WA, United States; ^3^ Graduate School of Applied and Professional Psychology, Rutgers University, New Brunswick, NJ, United States

**Keywords:** ADHD, working memory, inhibition, experimental psychopathology, anxiety

## Abstract

**Introduction:**

Children with ADHD demonstrate difficulties on many different neuropsychological tests. However, it remains unclear whether this pattern reflects a large number of distinct deficits or a small number of deficit(s) that broadly impact test performance. The current study is among the first experiments to systematically manipulate demands on both working memory and inhibition, with implications for competing conceptual models of ADHD pathogenesis.

**Method:**

A clinically evaluated, carefully phenotyped sample of 110 children with ADHD, anxiety disorders, or co-occurring ADHD+anxiety (*M_age_* =10.35, 44 girls; 69% White Not Hispanic/Latino) completed a counterbalanced, double dissociation experiment, with two tasks each per inhibition (low vs. high) x working memory (low vs. high) condition.

**Results:**

Bayesian and frequentist models converged in indicating that both manipulations successfully increased demands on their target executive function (BF_10_>5.33x10^8^, *p*<.001). Importantly, occupying children’s limited capacity working memory system produced slower response times and reduced accuracy on inhibition tasks (BF_10_>317.42, *p*<.001, *d*=0.67-1.53). It also appeared to differentially reduce inhibition (and non-inhibition) accuracy for children with ADHD relative to children with anxiety (BF_10_=2.03, *p*=.02, *d*=0.50). In contrast, there was strong evidence *against* models that view working memory deficits as secondary outcomes of underlying inhibition deficits in ADHD (BF_01_=18.52, *p*=.85).

**Discussion:**

This pattern indicates that working memory broadly affects children’s ability to inhibit prepotent tendencies and maintain fast/accurate performance, and may explain the errors that children with ADHD make on inhibition tests. These findings are broadly consistent with models describing working memory as a causal mechanism that gives rise to secondary impairments. In contrast, these findings provide evidence *against* models that view disinhibition as a cause of working memory difficulties or view working memory as a non-causal correlate or epiphenomenon in ADHD.

## Introduction

Impaired performance on executive function tests is well established in children, adolescents, and adults with attention-deficit/hyperactivity disorder (ADHD; e.g., 1, 2). Thus, it is not surprising that most contemporary neurocognitive/behavioral models of ADHD make predictions regarding the role of executive dysfunction in the etiology, pathophysiology, and/or recovery from the disorder (3, 4). As the two primary executive functions in school-aged youths (5), working memory and/or inhibitory control have garnered particular attention. They have been proposed to reflect core (causal) underlying neurocognitive deficits in ADHD (6–9), non-causal correlates of ADHD that may nonetheless aid in developmental recovery from the disorder (10), secondary outcomes of other core deficits (11, 12), and/or epiphenomenal difficulties that neither cause ADHD nor affect symptom expression/persistence (13). Among theoretical models that conceptualize one or both executive functions as core influences on ADHD symptom expression/diagnostic status, disagreement remains regarding the extent to which (a) underlying working memory deficits are responsible for poor performance on inhibitory control tests (e.g., 14), (b) underlying inhibitory control deficits are responsible for poor performance on working memory tests (e.g., 6), and/or (c) deficits in working memory and inhibitory control reflect correlated but relatively independent impairments (e.g., 15). Using a double dissociation design, the current study is among the first to experimentally manipulate both working memory and inhibitory control demands while concurrently measuring the effects of each executive function manipulation on performance on tests intended to measure the other executive function. In other words, the current study experimentally tests whether occupying children’s limited capacity working memory system by adding complex span-style recall demands disrupts inhibitory control performance. Concurrently, it also experimentally tests whether depleting inhibitory resources via a Stroop interference paradigm disrupts working memory performance in a carefully phenotyped clinical sample of children with ADHD, anxiety disorders, and co-occurring ADHD + anxiety disorders.

### Working memory and inhibitory control in ADHD

Executive functions are correlated but distinguishable neurocognitive processes that facilitate goal directed behavior and problem solving (16, 17). There are a plethora of executive function models spanning cognitive, behavioral, neurological, and sociocultural domains. Among these models, factor analytic and theoretical work provides significant support for models that include two primary executive functions in middle childhood: working memory and inhibitory control (for review, see 5). Set shifting, or cognitive flexibility, reflects the third core executive function, but generally does not emerge as a unique executive function until late adolescence or early adulthood (18, 19). *Working memory* refers to processes involved in the updating, dual-processing, and serial/temporal reordering of information held in short-term memory (20–23). *Inhibitory control* refers to processes that facilitate one’s ability to stop an ongoing response in the context of goal-directed behavior (24, 25).

Impairments on tests intended to measure working memory and inhibitory control are well established in pediatric ADHD, with meta-analytic effect sizes ranging from Cohen’s *d*=0.69-0.74 for working memory (and potentially as high as *d*=2.01-2.15 based on construct-valid working memory tests; 2, Kofler et al., in press^1^) to *d*=-0.03 to 0.63 for inhibitory control (24, 26–28). Cohen’s *d* is a measure of the magnitude of between-group differences, and is interpreted as: small (*d*=0.20), medium (*d*=0.50), or large (*d*=0.80). Similarly, heterogeneity estimates suggest that 62-85% of children with ADHD exhibit working memory deficits and 21-46% have impairments in inhibitory control (for review, see 29). Relevant for the present experiment, very few studies have controlled for one executive function while estimating the extent to which children with ADHD have impairments in the other executive function – an important limitation given their moderate intercorrelations (*r*=.43; 30) and disagreement among influential conceptual models regarding the primacy and relevance of these neurocognitive functions for explaining the ADHD phenotype (**Table 1**).

**Table 1:** Etiological models of attention-deficit/hyperactivity disorder (ADHD): Predictions regarding working memory and/or inhibitory control.

Model	Model description of ADHD	Model predictions for the current experiment	Representative publications
Basal Ganglia	A model of reinforcement learning which posits that the basal ganglia are responsible for a dynamic gating mechanism that selectively updates the contents of working memory in the prefrontal cortex; environmental reinforcement of updating then “critiques” the gating of the basal ganglia for improved future performance via dopaminergic signals. The model posits that reduced striatal dopamine is responsible for working memory and motivational deficits that are observed in ADHD.	Working memory and response inhibition are viewed as distinct and independent of one another. Observed deficits in working memory are posited as secondary to reduced striatal dopamine, whereas deficits in response inhibition are believed to be secondary to cortical noradrenergic dysfunction. In computational models, sequelae resulting from the dysfunction of dopamine and noradrenaline were independent of one another. If difficulties on working memory and inhibition tests are due to different neurotransmitter deficiencies, it stands to reason that increasing demands on one executive function would not be expected to impact performance on tasks intended to measure the other executive function.	Frank et al. (31, 32); Frank & O’Reilly (33)
Behavioral Inhibition	A core deficit model wherein deficits in behavioral inhibition (stopping pre-potent/ongoing responses and interference control) result in deficits in working memory and three other areas that collectively result in ADHD behavioral symptoms.	Increasing inhibition demands are expected to differentially affect children with ADHD, reflecting their core deficit in this ability. Working memory difficulties are viewed as an outcome of underlying inhibition deficits; therefore, the model would predict that increasing inhibition demands would differentially impact working memory performance for children with ADHD. In contrast, increasing working memory demands should not affect inhibition performance because working memory difficulties are ‘downstream’ of the core inhibition deficits.	Barkley (6, 34)
Cognitive Neuroenergetic	Decreased ATP production and inadequate lactate supply from deficient astrocyte functioning causes the behavioral features of inefficient and inconsistent performance in individuals with ADHD.	Predictions consistent with Functional Working Memory Model, but views compromised working memory as a secondary outcome of energetic insufficiency that becomes apparent with increases in processing and effort demands; this energetic insufficiency may directly affect other processes without mediation by working memory. Also predicts that ADHD is not characterized by a specific deficit in inhibitory control, but instead that performance is impacted across the board (including on inhibition tests) for children with ADHD due to dysregulated attention/energetic processes. Increasing demands on inhibition processes would not be expected to affect working memory performance because reduced performance on inhibition tests is viewed as attributable to underlying energetic insufficiency/attentional lapses rather than reflective of inhibition difficulties. Predictions regarding working memory are less clear: The model interprets increases in working memory load as a proxy for increases in effort and computational demands, which reveal the impact of inadequate energetic resources, while also positing that working memory difficulties are an outcome rather than causal factor in ADHD. Thus, the model would seem to predict that the current study’s working memory manipulation would either produce downstream difficulties on inhibition tests *or* that this manipulation would not further impact inhibition test performance beyond the existing impact of insufficient energetic/effort resources.	Sergeant (35); Killeen et al. (36); Killeen (37)
Default Mode Network	A multiple pathway model that hypothesizes that disruptions in cortico-striato-thalamo-cortical neuroanatomical circuitry–consisting of ‘hot’ and ‘cool’ regions–contribute to functional behavioral and cognitive differences in ADHD. Predictable oscillations in default mode (resting state) neural networks interfere with task-oriented neural processing, producing periodic lapses of attention.	Neither working memory nor inhibition deficits are viewed as causal factors in ADHD. Instead, the model predicts that default mode interference competes with task-related processing, causing attentional lapses that lead to increased variability and thus reduced performance on all types of tests, including working memory and inhibitory control tests. Does not imply a relation between working memory and inhibition. If difficulties on both types of tests are secondary to underlying default mode network intrusions, it stands to reason that increasing demands on one executive function would not be expected to impact performance on tasks intended to measure the other executive function.	Sonuga-Barke & Castellanos (38)
Dopamine Transfer Deficit	A neurobiological model that predicts that the anticipatory firing of dopamine neurons, which normally occurs in anticipation of rewards, is reduced or absent in ADHD. This leads to more rapid extinction of unreinforced behaviors (as opposed to slower extinction in the dynamic developmental model) and diminished partial reinforcement effect, which then contributes to impaired learning and motivation that would explain some of the core ADHD symptoms.	The model does not make specific predictions regarding working memory or inhibition. However, the authors posit that processing global contingencies (i.e., longer-term history of reinforcement) may involve working memory as it is a “higher integrative function”. Does not imply a relation between working memory and inhibition. Given that the model does not account for, or only peripherally discusses, inhibition and working memory, the model provides no expectations that increasing demands on one would affect performance on tests of the other.	Tripp & Wickens (39)
DSM-5 Clinical Model	Attention problems and hyperactivity/impulsivity reflect the disorder’s core deficits. Neurocognitive deficits may be present in a variety of areas, including in working memory and response inhibition, but are not described as core causal factors and it is specifically noted that tests of these abilities are not sufficiently sensitive or specific to serve as diagnostic indices.	Given that working memory and inhibitory control deficits are seen as peripheral rather than causal features, the model provides no expectations that increasing demands on one would affect performance on tests of the other.	DSM-5-TR APA (40)
Dynamic Developmental	A core deficit model that hypothesizes that reduced dopaminergic functioning causes narrower reinforcement gradients and altered extinction processes in normal behavior-consequence relationships. These deficient dual processes contribute to core ADHD symptoms and behavioral variability, which vary based on context, task, and function.	Failure to inhibit responses (disinhibition) reflects the behavioral manifestation of a pattern of inconsistent behavior-response associations affected by deficient reinforcement/extinction mechanisms which, in turn, disrupt the accumulation of simple behavioral response units into more complex and functional response chains. Model does not discuss working memory specifically, but rather posits that attentional deficits/variability also lead to poor executive functioning (behavioral planning) broadly. Given that the model does not account for working memory, and that inhibitory control deficits are seen as peripheral rather than causal features, the model provides no expectations that increasing demands on one would affect performance on tests of the other.	Sagvolden et al. (12)
Extended Temporal Difference	A neurocomputational behavioral model derived from dopamine-driven temporal-difference learning (i.e., reinforcement learning) to explain impulsive behavior in ADHD. This model proposes that performance on a delayed response task depends on four contextual factors that influence preference for immediate rewards, including brittleness (predictability; the extent to which behavior is based on learned responses), action bias (preference for action over inaction), learning rate (rate of behavior change), and the discount factor (a predicable reward delivered in the future is less valuable than the same reward delivered immediately).	This model predicts that variation in simple learning and behavioral parameters predict increased difficulty with inhibition, which may reflect deficient signaling of reward-prediction error and hypo- and hyperfunctioning of the dopamine signal to rewards. On complex cognitive tasks, poor performance on long trials may be attributable to working memory deficits, whereas poor performance on short trials may be attributable to inhibitory control deficits. Given that working memory and inhibitory control deficits are seen as linked with different types of difficulties, the model provides no expectations that increasing demands on one would affect performance on tests of the other.	Williams & Taylor (41); Williams & Dayan (42); Durston et al. (43)
Functional Working Memory	A core deficit model that views ADHD symptoms as phenotypic/behavioral expressions of the interaction between neurobiological vulnerability & environmental demands that overwhelm impaired working memory. Associated features of ADHD, including inhibition difficulties, arise through direct effects of impaired working memory, or indirect effects of impaired working memory through its impact on core behavioral symptoms.	Increasing working memory demands are expected to differentially affect children with ADHD, reflecting their core deficit in this ability. Difficulties on inhibition tests are viewed as an outcome of underlying working memory deficits; therefore, the model would predict that increasing working memory demands would differentially impact inhibition task performance for children with ADHD. In contrast, increasing inhibition demands should not affect working memory performance because inhibition difficulties are ‘downstream’ of the core working memory deficits.	Rapport et al. (14, 44) Alderson et al. (45); Kofler et al. (29)
Moderate Brain Arousal	A neurocomputational model that views ADHD-related attention and cognitive difficulties, including working memory and inhibition deficits, as a function of low levels of baseline dopamine. Within this theory, environmental noise (e.g., white noise) can compensate for a hypofunctional dopamine system via increasing internal neural noise, which in turn improves cognitive functioning. Cognitive performance and dopamine transmission is further posited to follow an inverted U-shaped curve, such that too low or high levels attenuate performance.	Given that too low or too high brain arousal states are hypothesized to reduce cognitive performance, increasing working memory and inhibition demands to an optimal moderate brain arousal state will result in the best cognitive performance. In other words, too low and high working memory and inhibition conditions are likely to result in worse cognitive performance. Increasing working memory load is posited to *improve* performance in individuals with ADHD as they go from a low to moderate brain arousal state, whereas excessive working memory load is posited to *impair* performance due to a high brain arousal state. Predictions for the current experiment are unclear because we included only two working memory levels and did not directly measure brain arousal state.	Sikstrom & Soderlund (46); Grace (47, 48); Seeman & Madras (49)
Optimal Stimulation	Hyperactive children are chronically under-aroused due to inadequate neurotransmission and/or a shift in the level of stimulation these children find to be optimal. A feedback model based on the assumption that response output functions homeostatically to regulate the level of stimulus input.	The model does not discuss working memory/inhibition or executive functions specifically, but makes prediction regarding complex tasks more generally. During early stages of task acquisition, the tasks’ novelty is thought to provide sufficient stimulation. When the task is learned, task stimuli are repetitive, and/or sustained attention is required, the stimulation provided by the task is insufficient and children with ADHD may need to augment their arousal levels by increasing their activity level or altering their attentional response. Whether this activity-generated stimulation interferes with successful task performance will depend on the attentional requirements of the task, the difficulty of the task, and the level of performance. For the current experiment, if we assume that the tasks are not novel (i.e., due to multiple practice rounds), it seems reasonable to assume that the model would predict performance difficulties across all four tasks that are secondary to interfering effects of increased physical movement and visual attention to task-irrelevant stimuli (e.g., looking around, increased verbalizations). It may be further hypothesized that the ‘word’ task would produce the least interfering behaviors (because it requires the least complex executive processing) and the stroop span condition would produce the most interfering behaviors because it might be viewed as the most ‘difficult’ (i.e., because it requires both working memory and inhibitory processes). However, because task ‘difficulty’ is a nebulous concept, the model does not provide testable predictions regarding the impact of working memory on inhibition performance or vice versa.	Zentall & Zentall (50)
Subcortical Deficit	A developmental model that hypothesizes that ADHD is caused by subcortical neural dysfunction that manifests early in ontogeny, remains relatively static throughout life, and is not associated with the remission of symptomatology. ADHD behavioral symptoms reflect unconsciously (i.e., non-prefrontally) mediated deficits in arousal and activation similar to those described by the Cognitive Energetic Model. Executive dysfunction does not cause ADHD symptoms, but developmental growth in executive functions facilitates recovery. Executive functions are viewed as compensatory – they are not causally related to the disorder.	Given that working memory and inhibitory control deficits are seen as non-causal, compensatory features, the model provides no expectations that increasing demands on one would affect performance on tests of the other.	Halperin & Schulz (10); Halperin et al. (51)
Tripartite Pathway	A multiple pathway/equifinality model in which ADHD symptoms are caused by deficits in one or more dissociable cognitive (behavioral inhibition, temporal processing) and/or motivational (delay aversion) processes. Heterogeneity model; ADHD symptoms attributable to inhibition, delay, and/or temporal processing deficits, each affecting some ADHD patients	Working memory is not viewed as a core, causal deficit, whereas inhibitory control reflects the core deficit for a subset of patients. Working memory is discussed as a correlate of all three causal components but the model does not directly discuss the direction of the relation between inhibitory control and working memory (although inhibition is highlighted as a causal factor and working memory is not). Overall, model predictions for the current experiment are generally consistent with the behavioral inhibition model, with the caveat that inhibitory control is a causal factor for only a subset of children with ADHD.	Sonuga-Barke et al. (8); Lambek et al. (52)
Updated Executive Function Model	An update to the Behavioral Inhibition model in which working memory is elevated from a mediator variable to a primary causal factor alongside inhibitory control.	Increasing working memory or inhibition demands are both expected to differentially affect children with ADHD, reflecting their core deficits in these abilities. Working memory and inhibitory control are viewed as correlated core deficits. Therefore, increasing demands on one core process would not be expected to impact performance on tasks intended to measure the other core process.	Barkley (53); Panah et al. (15)
Variability Trait	Childhood ADHD behaviors attributed to excessive variability, both in rate and magnitude of change, in arousal level and reactivity; excessively inconsistent arousal and reactivity result in problems in sustained attention, performance, and social behavior. Excessive variability in autonomic, electrocortical and behavioral response underlies impairments in attention, performance, and social behavior.	Reduced performance on working memory and inhibition tests (as well as all kinds of tests) reflect inconsistent performance; a ‘third variable’ model in which increasing working memory or inhibition demands would not be expected to affect performance on tests of the other ability because both are outcomes of excessive trait inconsistency.	Hicks et al. (54)

BI, behavioral inhibition; CE, central executive; WM, working memory.

A partial exception to this methodological critique comes from a study finding that covarying working memory eliminated ADHD/neurotypical between-group differences in inhibition, whereas covarying inhibition produced only a small reduction in ADHD/neurotypical working memory differences (45). Similarly, a recent randomized control trial (RCT) found that targeted training of inhibitory control did not produce improvements in working memory for children with ADHD, whereas targeted training of working memory produced superior improvements in inhibitory control relative to the active, credible neurocognitive control training – albeit only on one of two inhibition tests (55). A similar pattern has also been found in training studies of healthy children as well as adults with borderline personality traits (but not healthy adults) – in each case, working memory updating training, working memory maintenance (short-term memory) training, and dual n-back training produced superior improvements on one of two inhibition tests relative to passive controls (56–58; cf. 59, 60).

These findings are generally consistent with *dual-mechanism* accounts of inhibitory control from the cognitive literature (61), which emphasize the impact of working memory capacity for resolving response competition (e.g., between the conflicting color and word dimensions in the Stroop task), maintaining task goals that are not sufficiently reinforced by the environment (62–64), and/or controlling attention to prevent intrusions from irrelevant distractors (65). Applied to ADHD, Rapport and colleagues (7, 14) have argued that inhibitory control difficulties are more parsimoniously viewed as an outcome of working memory difficulties rather than a cause, at least in part because “inhibition is a reaction to external stimuli that must first gain access to and be evaluated within working memory” (45, p. 498). Together, these studies appear to provide preliminary support for conceptualizing difficulties on inhibition tests among children with ADHD as, at least in part, artifacts of their underlying working memory difficulties (7).

In contrast, others have argued that inhibitory control deficits in ADHD lead to secondary deficiencies in working memory because inhibition ‘sets the occasion’ for working memory to function by providing the necessary delay for it to occur (e.g., 6). In this view, inhibition is conceptualized as a limited resource that will be depleted when external demands exceed that resource (30, 66–68; cf. 69–71). When inhibitory resources are depleted, task-irrelevant information is able to gain access to the working memory system. In turn, this produces interference effects that impair maintenance of task goals and rehearsal of to-be-recalled test items (45, 72). Thus, we would expect children with ADHD to have fewer inhibitory resources available to maintain task goals and protect stimuli in working memory, particularly when those inhibitory resources are depleted by imposing interference demands (73). This view is broadly consistent with *depletion* accounts of inhibitory control from the social psychology literature, which describe inhibitory control as a limited, consumable resource that, when depleted (e.g., through engagement with inhibition tasks as in the current experiment) will not be available to support additional executive processing (67, 68).

In partial support for this view, a recent RCT with healthy adults found that adding inhibition demands to an *n*-back training protocol resulted in superior improvements in working memory updating and short-term memory recall relative to a passive control group. However, interpreting these effects as attributable to inhibition training is challenging because the training groups did not show improved inhibition performance relative to the passive control group (60). Similarly, Alderson and colleagues (30) conducted, to our knowledge, the only relevant ADHD experimental/dual-task manipulation study to date. They found that increasing inhibition demands disrupted *n*-back memory performance for children with and without ADHD, whereas increasing *n*-back memory load failed to affect inhibition processes (30). These findings may suggest that inhibition is upstream from working memory in children with and without ADHD, because adding inhibitory demands created a bottleneck that disrupted the cognitive resources available for working memory processing. Interestingly, however, adding inhibition demands appears to have had a larger effect on the neurotypical group than the ADHD group (i.e., between-group differences were significant for the 1-back task but not the 1-back + stop-signal dual task due to differentially reduced performance in the neurotypical control group), calling into question the extent to which inhibition is a causal factor in ADHD-specific working memory difficulties. Further, despite the elegant experimental design, Alderson et al. (30) pointed out that their high working memory condition (2-back) was simply too difficult for all children (i.e., performance at/below chance levels). This may suggest that the working memory manipulation may have been less successful than intended and limit conclusions regarding working memory’s impact on inhibitory control functioning.

In addition to mixed evidence supporting each executive function as an upstream driver of ADHD-related difficulties with the other executive function, there is also evidence suggesting that they may reflect independent impairments in ADHD. For individuals with ADHD specifically, Panah et al. (15) directly tested the Barkley inhibition/updated executive function models. They found that the structural equation model with working memory and inhibition as correlated predictors provided a better fit to the data relative to the model in which working memory was modeled as an outcome of inhibition (15), suggesting that these may be relatively independent impairments in ADHD. Similarly, Kofler et al. (9) reported that only 17% of children with ADHD have impairments in both inhibition and working memory (vs. 46% who have working memory but not inhibition deficits, and only 11% who have inhibition deficits but not working memory deficits). Karalunas et al. (74) also found that only 13% of children with ADHD have stable impairments in both inhibition and working memory (vs. 44% who have stable working memory but not inhibition deficits, and only 5% who have stable inhibition deficits but not working memory deficits).^2^ The similarity in these estimates is striking, especially given that the former was based on cross-sectional factor-analytic estimates using multiple tests per construct and the latter was based on a single test per construct with latent class growth analysis from a 3-year longitudinal study. Together, these findings suggest that only a small minority of children with ADHD have impairments in both working memory and inhibitory control, and thus appear to support models conceptualizing them as relatively independent impairments in ADHD. Finally, it is also possible that working memory and inhibitory control exert bidirectional effects on each other and/or that depleting resources on either process would impair performance on tests of the other process (75). However, to our knowledge, no current ADHD conceptual models make this prediction.

### Working memory and inhibitory control in anxiety

As noted above, children with anxiety disorders served as the clinical comparison group (compared with children with ADHD and ADHD+anxiety) in the current study. This was a pragmatic decision because recruitment of a typically developing control group was not feasible due to funding constraints. Thus, a commentary on the relation between anxiety and executive functioning is warranted. Interestingly, whereas several theoretical models conceptualize executive function deficit(s) as underlying *causes* of ADHD (e.g., 29), they tend to be viewed as *outcomes* of anxiety disorders or involved in the maintenance of anxiety symptoms (76–78). However, studies of executive functioning in children with anxiety disorders have been surprisingly mixed. Regarding inhibitory control, meta-analytic evidence indicates that anxiety disorders are not associated with impairments (*ns*; 79) or are associated with small magnitude impairments (*d*=-0.31; 80) that are significant based on analysis of response times (*d*=-0.27) but not accuracy data (*ns*; 81). Regarding working memory, recent meta-analyses diverge in documenting a small magnitude impairment in children with anxiety disorders (*d*=-0.24; 82), no significant impairment (*ns*; 80), or no significant impairment based on response time data (*ns*) but a small, significant *strength* based on accuracy data (*d*=0.38; 81) that was also found in a recent empirical study controlling for ADHD status (*d*=0.19; 83). However, when examined, effect sizes tended to be similar across anxiety disorder categories, anxiety severity, and/or state versus trait anxiety (80–82), at least for the diagnoses included in the current study (please see *Method* section below).

Applied to the current study’s outcome data, these meta-analytic estimates suggest that our use of an anxiety disorder group as the clinical comparison group may produce a slight overestimate of ADHD-related working memory deficits (based on accuracy data). It may also either not affect (accuracy data) or produce a small underestimate (response times/RTs) of ADHD-related inhibition deficits. In contrast, co-occurring anxiety disorders do not appear to affect estimates of working memory deficits in children with ADHD but may exert a small protective effect by reducing the magnitude of inhibition deficits in co-occurring ADHD+anxiety relative to ADHD-only groups by *d*=0.14-0.41 across meta-analyses (79, 83, 84). Thus, estimates of ADHD-related impairments should be interpreted with the clinical nature of the comparison group in mind.

### Current study

Taken together, children with ADHD demonstrate difficulties on tasks intended to measure inhibitory control and working memory. However, it remains unclear whether this pattern reflects multiple, distinct impairments or may be more parsimoniously accounted for by a single deficit that broadly affects performance (85, 86). The current study uses a double dissociation design to test competing model predictions regarding the directionality of these impairments in ADHD. Support for working memory-focused models would include significant reductions in inhibitory control performance when working memory demands are experimentally induced (14, 45). In contrast, support for behavioral inhibition-focused models (e.g., 6, 8) would include significant reductions in working memory recall as inhibitory control demands were experimentally increased. Alternatively, support for correlated core deficit, non-causal, recovery, and epiphenomenal models of executive functions in ADHD would include significant evidence *against* changes in one executive function when demands on the other executive function were experimentally increased. Finally, as noted above to our knowledge no ADHD conceptual models predict bidirectional causality (i.e., that increasing working memory demands would disrupt inhibitory control performance *and* increasing inhibitory control demands would disrupt working memory performance).

## Method

### Transparency and openness

We report how we determined our sample size, all data exclusions (if any), all manipulations, and all measures in the study. Data were analyzed using JASP v.0.17.2.1 (87). All measure inclusion/exclusion decisions and analytic plans were made *a priori*, prior to accessing the data; however the study was not publicly pre-registered. Data/code and results output are available on our Open Science Framework website: https://osf.io/gts6x/. Descriptions in the *Participants*, *Group Assignment*, *Procedure*, *Overview*, *IQ/SES*, and *Bayesian* sections below are reproduced/adapted from our standard research/clinic recruitment and testing protocols licensed under CC BY 4.0.

### Participants

The sample comprised 110 children (44 girls) ages 8 to 13 years (*M*=10.35, *SD*=1.30) from the southeastern United States recruited by or referred to the Children’s Learning Clinic (CLC) through community resources (e.g., pediatricians, schools, self-referral) between July 2018 – March 2020 and October 2021 – August 2022 for participation in a larger study examining links between children’s neurocognitive, attentional, and behavioral functioning. The gap reflects the COVID-19 shutdown followed by our COVID-19 health and safety protocol that temporarily reduced our research battery. The CLC is a research-practitioner training clinic that conducts developmental and clinical child research and provides no-cost diagnostic, psychoeducational, and treatment services. Its client base consists of children with suspected behavioral, learning, or emotional difficulties. Sample ethnicity was mixed and included 76 White Not Hispanic (69.1%), 16 Black or African American (14.5%), 6 Hispanic or Latino (5.5%), and 12 multiracial (10.9%) children.

As noted above, funding constraints prevented us from recruiting a typically developing sample (those without suspected psychological disorders) for the current experiment. Our recruitment strategy thus emphasized participation of children in need of clinical evaluation who were, and were not, suspected of having ADHD. Recruitment of a non-ADHD clinical sample allows for more robust control for the presence of these co-occurring diagnoses in the ADHD group (i.e., it allows us to draw stronger conclusions about processes implicated in ADHD specifically as opposed to processes that may appear to be impaired in ADHD due to the confounding influence of co-occurring conditions; 88). Additionally, given the large number of studies examining working memory and/or inhibitory control in ADHD versus neurotypical samples, our inclusion of a clinical comparison group can be considered a strength because it extends prior work by testing the extent to which ADHD-related impairments in executive functioning are evidenced above and beyond difficulties attributable to another common form of child psychopathology. Parents/children gave informed consent/assent; Florida State University Institutional Review Board approval was obtained/maintained.

### Group assignment

All families completed a comprehensive psychoeducational evaluation that included detailed, semi-structured parent clinical interviewing (K-SADS; 89), parent and teacher rating scales (e.g., ADHD-RS-5, BASC-3; 90, 91), and norm-referenced child internalizing disorder screeners. Additional measures were administered based on clinical judgment and presenting problems to facilitate differential diagnosis and accurately capture clinical comorbidities (e.g., semi-structured child clinical interviews, additional testing). Parents received a psychoeducational report; children picked a toy (≤$5) from our prize box.

Three clinical groups of children participated in the current experiment: children with ADHD (without anxiety), children with ADHD + co-occurring anxiety (ADHD+ANX), and children with anxiety (without ADHD). Fifty-nine children (21 girls) met all of the following criteria and were diagnosed with ADHD (without anxiety) based on the comprehensive psychoeducational evaluation: (1) DSM-5 diagnosis of ADHD combined (*n* = 38), inattentive (*n* = 20), or hyperactive/impulsive (*n =* 1) presentations by the CLC’s directing clinical psychologist and multidisciplinary team based on K-SADS and differential diagnosis considering all available clinical information indicating onset, course, duration, and severity of ADHD symptoms consistent with the ADHD neurodevelopmental syndrome; (2) borderline/clinical elevations on at least one parent and one teacher ADHD subscale (i.e., > 90th percentile); and (3) current impairment based on parent report. Children with any current ADHD presentation specifiers were eligible given the instability of ADHD presentations (92–94).

The ADHD+ANX group was comprised of an additional 28 children (11 girls) who met criteria for ADHD based on the criteria above (18 combined, 9 inattentive, 1 hyperactive/impulsive presentation), and also met criteria for one or more anxiety disorders (11 generalized, 10 social, 2 separation, 6 other specified, 5 specific phobia [dark]).^3^ Finally, the ANX (without ADHD) group was comprised of 23 children (12 girls) who completed the same comprehensive psychoeducational assessment and did not meet criteria for ADHD, but met criteria and were diagnosed with one or more anxiety disorders (9 generalized, 7 social, 1 separation, 8 other specified, 1 specific phobia).

Several children in each group also met criteria for common clinical/learning disorders beyond ADHD and/or anxiety based on the comprehensive psychoeducational evaluation, including oppositional defiant disorder (6.4%)^4^, autism spectrum disorders (13.6%), depressive disorders (6.4%), and specific learning disorders (20.0%). To improve generalizability given that comorbidity is the norm rather than the exception for children with ADHD (95), these children were retained in the sample. As described below, the distribution of these additional syndromes was generally evenly distributed among the three clinical groups. Psychostimulants (*N_prescribed_* = 18) were withheld ≥24 hours prior to neurocognitive testing.

None of the children presented with gross neurological, sensory, or motor impairments that would preclude valid test administration, history of seizure disorder, intellectual disability, psychosis, or non-stimulant medication that could not be withheld for testing.

### Procedure

This experiment was embedded within a larger battery of counterbalanced executive and non-executive research tasks. Study procedures were identical to those reported in the Kofler et al. (85) experiment, with new tasks and a non-overlapping sample. Testing occurred during a larger battery of two, 3-hour sessions. Tasks were counterbalanced within/across sessions to minimize order/fatigue effects. Children whose counterbalancing resulted in them completing one or more of the low memory tasks after previous exposure to one or more of the high memory task variant(s) described below were explicitly told not to remember the colors. Children received brief breaks after each task and preset longer breaks every 2-3 tasks to minimize fatigue. Performance was monitored by an examiner stationed just outside the testing room to provide a structured setting while minimizing performance improvements associated with examiner demand characteristics (96).

### Experiment overview

We created a dual dissociation experiment using four computerized tasks to experimentally address the directionality of inhibitory control and working memory deficits in ADHD (**Table 2**). Two of the four tasks were working memory complex span tasks, adapted for children based on principles underlying the classic reading span and counting span tasks (97), one with low inhibition demands (*word span* task = low inhibition, high working memory) and one with high inhibition demands (*stroop span* task = high inhibition, high working memory). The remaining two tasks omitted the memory demands but were otherwise identical to the complex span tasks: one with low inhibition demands (*word* task = low inhibition, low working memory) and one with high inhibition demands (*stroop* task = high inhibition, low working memory).

**Table 2:** Fully-crossed experimental design overview.

		Working Memory Demands	
		**Low**	**High**
Inhibitory Control Demands	**Low**	**Word-Color (‘Word’) Task** Children identify the font color of each neutral (non-color) word	**Word-Color Span (‘Word Span’) Task** Identical to the Word-Color Task, with the addition of a recall phase after every 6 stimuli.
	**High**	**Stroop Color-Word (‘Stroop’) Task** Children identify the font color of each color word. On 20% of trials, the font color and the printed word do not match, creating interference effects.	**Stroop Color-Word Span (‘Stroop Span’) Task** Identical to the Stroop Task, with the addition of a recall phase after every 6 stimuli.

### Task overview

**Figure 1:**
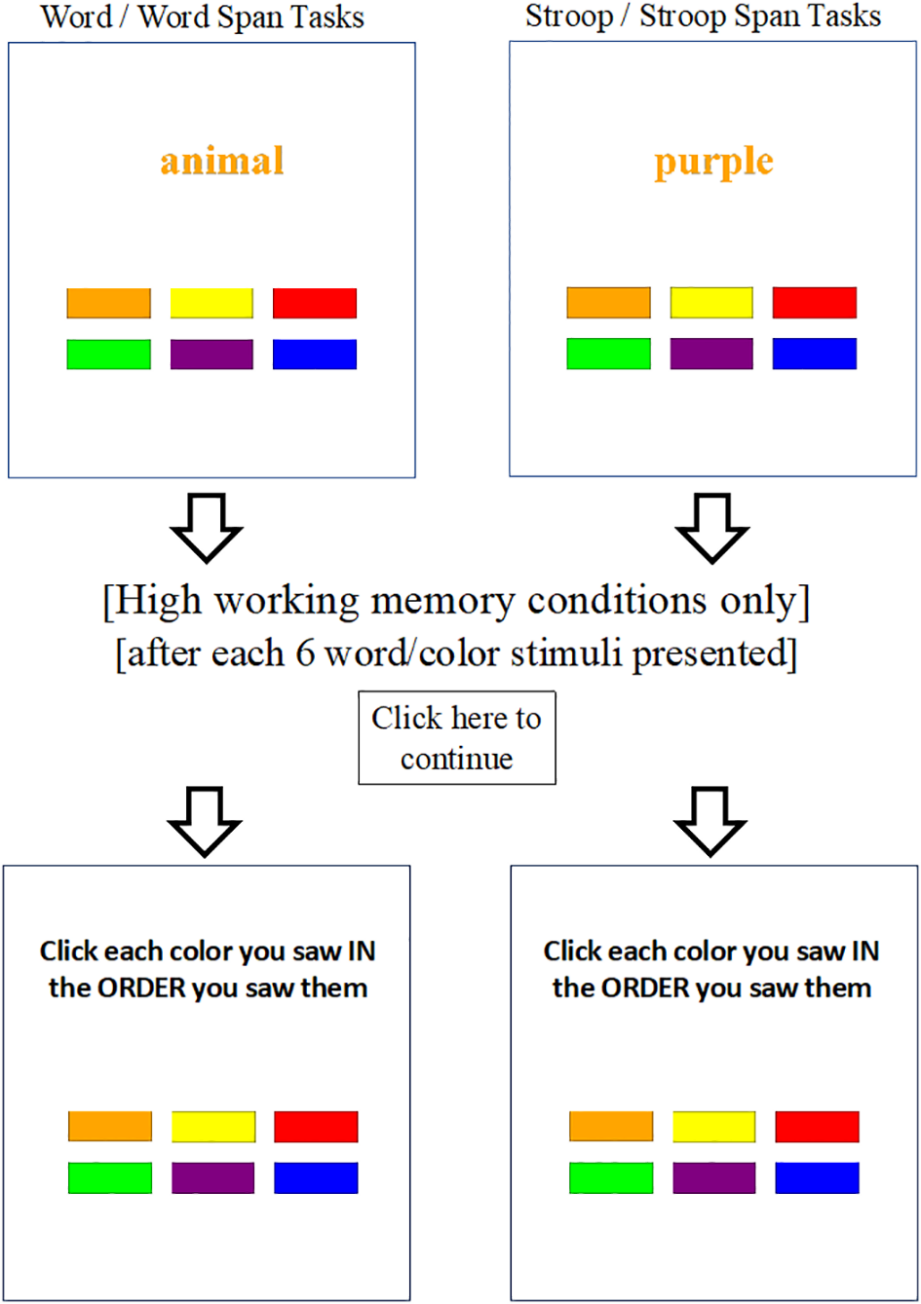
Fully-crossed experimental manipulation of working memory and inhibition demands. Each of the 4 counterbalanced tasks presented 150 stimuli, randomly without replacement. High inhibition (i.e., stroop) conditions presented color words and featured an 80:20 ratio of congruent (printed word matches the font color) and incongruent (printed word does not match the font color) trials to maximize prepotency/inhibition demands on the critical incongruent trials. Low inhibition (i.e., word/non-stroop) conditions were identical to the high inhibition versions except they presented neutral, non-color words. Each of the two low/high working memory task pairs were identical except for the omission or addition of concurrent working memory demands. Words/icons outside the boxes were not shown on screen, but are included here to illustrate differences across the four otherwise identical experimental tasks. Note: ‘animal’ and ‘purple’ in this example are both printed in orange font color (correct response = clicking orange response box for both). Response boxes from left to right are (top row) orange, yellow, red, (bottom row) green, purple, blue.

One hundred and fifty (150) color/word stimuli were presented for each task. In all conditions, children were instructed to always respond to the font color (the color that the word is printed in) and to ignore the meaning of the word. None of the children presented with parent-reported color blindness, and as described below practice trials were completed to ensure children could correctly identify/name the font colors and fluently read the color words. Children responded by clicking colored response boxes on the screen (**Figure 1**). All tasks were self-paced with a pre-programmed break halfway through. Our *a priori* plan called for removal of anticipatory responses (trial RTs < 150 ms); however, no cases were identified. Internal consistency reliability was excellent for the current sample for all 4 tasks (α=.92-.95).

#### Practice trials

On-screen performance feedback (correct, incorrect) was provided for every practice response/trial. All task variants began with two practice phases (6 trials each; 80% correct required): In the first practice phase (color naming), children were shown colored rectangles, one at a time, and instructed to verbally name the color. For the second practice phase (color word reading), children were shown color words in black font and asked to read the word and click the response box that matched the word’s meaning (e.g., see the word “red” and click the red colored response box).

For the low inhibition tasks (i.e., non-stroop variants: *word*, *word span*), the third practice phase presented neutral, non-color words (e.g., the word “the” printed in red font) and children were required to respond based on the printed font color while ignoring the word meaning (6 trials; 80% correct required). For the high inhibition tasks (i.e., stroop variants: *stroop*, *stroop span*), the third practice phase presented color words printed in incongruent colors (e.g., the word “red” printed in blue font) and children were required to respond based on the printed color while ignoring the word meaning (6 incongruent trials; 80% correct required).

For the high working memory variants *(word span*, *stroop span*), a final practice phase introduced the memory component. This practice phase mirrored the stroop (for *stroop span*) or non-stroop (for *word span*) third practice phase described above, except this time children were instructed to remember the colors in the order presented. For these high working memory conditions, practice trials at memory set 4 were terminated after two 100% correct recall trials.

#### High inhibition, low working memory

For the stroop color-word identification task (i.e., *stroop* task), children were presented with color words (red, orange, yellow, green, blue, purple) printed in font colors that either matched or did not match the meaning of the color word, one at a time, on the computer monitor. The task’s inhibition demands occur because of the overlearned, automatic tendency to read words, combined with task instructions to ignore the word’s meaning and instead respond based on the word’s font color. The task’s well-documented interference effects occur when the color word is printed in a color different than the word’s meaning (e.g., the word ‘red’ printed in blue font), requiring inhibitory control processes to stop the automatic word reading while prioritizing the less automatic color recognition (98).

The stroop task was considered ideal for inducing inhibition processes in the current experiment (99) because it is thought to place demands on both the response inhibition and interference control subcomponents of the inhibitory control construct (100). The ‘experimental stroop’ was preferred over the classic version included in standardized neuropsychological test batteries. This decision was made because the latter have been criticized for presenting blocks of all incongruent trials, which reduces prepotency and thus evokes lower demands on the inhibitory control process of primary interest (99, 101). Thus, as recommended by Snyder et al. (99) and following Kane and Engle (61; Experiment 4), our ‘experimental stroop’ task featured an 80:20 ratio of trials that did not vs. did require participants to inhibit their automatic/prepotent response to reading the color word (24, 99). Thus, on 120 of the 150 trials (80%), the printed word and the word’s font color matched (*congruent trials*; e.g., the word ‘red’ printed in red font). On the critical 30 *incongruent trials* (20%), the printed word and its font did not match (e.g., the word ‘red’ printed in blue font).

Following Kane and Engle (61), for analytic purposes, 30 of the congruent trials were labeled as ‘critical’ congruent trials, and the remainder were labeled as ‘filler’ trials. This labeling occurred in the software backend for scoring purposes; there was no observable distinction between filler and critical congruent trials for participants. All 30 incongruent trials were ‘critical’ trials. The dependent variables for the stroop task were median response times to correct trials (RT; milliseconds) and accuracy (% correct), separately for the incongruent and critical congruent trials. Median RT was used *in lieu* of mean for all tasks given the well documented variability in reaction times in children with ADHD that are attributable to positive skew (102).

#### Low inhibition, low working memory

The word-color identification task (i.e., *word* task) was identical to the stroop task except that neutral, non-color words were presented. As with the stroop task, children were instructed to respond based on the font color of the word. The same colored response boxes were used on all tasks (**Figure 1**); thus, there were no response options related to the meaning of these non-color words (i.e., no interference effects are expected because reading the words does not activate any of the available response options). The neutral words were selected to match the letter length of the stroop condition’s color words (the/red, animal/orange, letter/yellow, house/green, word/blue, number/purple). Following Kane and Engle (61), thirty of the stimuli were randomly labeled as ‘critical congruent’ and an additional 30 were randomly labeled as ‘critical incongruent’ in the software backend to match the stroop task for scoring and analysis purposes. The dependent variables for the word task were median response times to correct trials (RT; milliseconds) and accuracy (% correct), separately for the ‘critical incongruent’ and ‘critical congruent’ trials.

#### High inhibition, high working memory

For the current experiment, we created a task that combined the experimental stroop task with classic complex span (dual-processing working memory) task design as described above (29, 97, 103). The stroop complex span task (i.e., *stroop span*) was identical to the stroop task except that a recall phase was inserted after every 6 color-word stimuli (25 total recall trials). During the recall phase, children were tasked with remembering and clicking the response boxes corresponding to the font colors that were presented, in the order that they were presented for that trial. Dependent variables are the same as those described for the stroop task, as well as recall accuracy (% of stimuli recalled correctly). Accuracy data based on recall of colors that were presented as congruent and incongruent stimuli were recorded separately to allow more nuanced examination of the extent to which color-word inhibition processes interfere with the encoding of to-be-recalled stimuli.

#### Low inhibition, high working memory

The word-color identification complex span task (i.e., *word span*) was identical to the stroop span task, except that it used the neutral, non-color words from the word task. Dependent variables are identical to the stroop span task, with ‘critical congruent’ and ‘incongruent’ stimuli defined randomly in the software backend as described above for the word task.

### Primary outcomes: working memory

The proportion of stimuli correct per trial (% recalled correctly) during the recall phases of the word span and stroop span tasks was used to assess working memory capacity as recommended (29, 97). Performance was assessed for each child separately for each of the two complex span tasks (word span, stroop span). By design, there was no recall phase during the low working memory conditions. Following Kofler et al. (29), scores from these conditions reflect initial encoding accuracy. In other words, the low working memory conditions control for encoding, because the high working memory conditions involve both encoding and working memory maintenance/recall (29, 97). As argued previously (29), we prefer the term “low” rather than “no” working memory because at least some working memory demands are likely involved in all tasks (e.g., maintaining rule sets, attentional control to task demands). As noted above, scores were computed separately for ‘congruent’ and ‘incongruent’ stimuli as defined above, which were both included in the statistical models as a within-subjects factor (Trial Type). Higher scores reflect better working memory.

### Primary outcomes: inhibitory control

Response times (median RTs to correct trials; milliseconds) and accuracy (% correct) during the primary color identification component of each task were used to assess the components of task performance that are compared to assess individual differences in inhibitory control. Thus, separate scores were recorded for incongruent trials and critical congruent trials as described above, and both were included in each statistical model as a within-subject factor (Trial Type). Smaller reductions in speed and/or accuracy during incongruent relative to congruent trials during the high inhibition tasks (reflected in the within-subject effect Inhibition Low/High x Trial Type Congruent/Incongruent interaction described below) reflect better inhibitory control.

### Intellectual functioning (IQ) and socioeconomic status (SES)

IQ was assessed using the 4-subtest Short Form of the *Wechsler Intelligence Scale for Children, Fifth Edition* (WISC-V) (104). SES was estimated using the Hollingshead scoring based on caregiver(s)’ education and occupation (105).

### Bayesian analyses

Both Bayes Factors (BF) and *p*-values are reported as recommended (106). Bayes Factors are included because they estimate the magnitude of support for both the alternative hypothesis and the null hypotheses, and are thus able to provide support for the null hypothesis rather than just failing to reject it (107). BF_10_ indicates how much more likely the alternative hypothesis (H_1_) is relative to the null hypothesis (H_0_). BF_01_ is the inverse of BF_10_ (i.e., BF_01_=1/BF_10_), and is reported when the evidence favors the null hypothesis. As recommended, we used the ‘test, then estimate’ method, such that we first tested whether an effect likely exists (via *p*-value/Bayes Factor) and then estimated the magnitude (effect size) for significant effects; when the evidence favors the null hypothesis, the most parsimonious effect size estimate is 0.0 (108).

### Data analysis overview

The current study used a fully-crossed 2x2 experimental design (within-subjects effects: Inhibition demands Low/High x Working Memory demands Low/High), with 3 groups (between-subjects effect: Group = ADHD, ADHD+ANX, ANX), and 2 outcomes per task (within-subjects effect: Trial Type = Incongruent, Congruent). We thus examined the study’s primary hypotheses via mixed-model ANOVAs, using both classical (frequentist) and Bayesian statistics. Following manipulation checks to ensure that each experimental manipulation successfully engaged its target executive function as intended, Tier 1 probed for effects of experimentally increasing inhibition demands on working memory performance (DV: percent correctly encoded or encoded+recalled). Tier 2 tested for effects of increasing working memory demands on inhibitory control performance, with one model for speed (DV: RT to correct trials) and one model for accuracy (DV: % correct). Exploratory analyses were conducted in Tier 3 to probe for alternative explanations for the obtained pattern of results.

## Results

### Power analysis

To our knowledge, power analysis for Bayesian repeated-measures ANOVA is not yet available. Power analysis with G*Power (v3.1; 109) based on traditional NHST, with alpha=.05, power=.80, 3 groups, and 8 measurements (the 4 tasks described above, with 2 variables from each task in each model) indicates that our *N*=110 can reliably detect within-group and interaction effects of *d*=0.20, and between-group effects of *d*=0.46 or larger. Thus, the study is sufficiently powered to address its primary aims.

### Preliminary analyses

Outliers ≥3 *SD* were winsorized relative to the within-group distribution (ADHD=1.6%, ADHD+ANX=2.0%, ANX=0.40% of data points). All parent and teacher ADHD symptom ratings were higher for the ADHD and ADHD+Anx groups relative to the (non-ADHD) ANX group as expected, with one exception (*p*=.06; **Table 3**). As shown in **Table 3**, the groups were equivalent or did not differ based on sex, SES, ethnicity, or co-occurring conditions including ASD, SLD reading, and SLD math. In contrast, all of the ODD cases were in the ADHD-only group, and the ANX group was slightly older than both the ADHD and ADHD+ANX groups, who were equivalent. As described below, sensitivity analyses indicated that the results were robust to control for age. This is the first reporting of data from any of these tasks for any children in the current sample, and none of the children in the current sample were included in any of our prior experimental studies.

**Table 3:** Sample and demographic variables.

Variable	ADHD (*n*=59)		ADHD+ANX (*n*=28)		ANX (*n*=23)		*p*	*BF_10_*	*BF_01_*	*Post-hocs*
	*M*	*SD*	*M*	*SD*	*M*	*SD*				
Sex (Girls/Boys)	21/38		11/17		12/11		.39, *ns*		5.17	–
Ethnicity (B/H/M/W)	10/4/6/39		2/1/5/20		4/1/1/17		.65, *ns*		277.30	–
Age	10.26	1.30	9.97	1.24	11.05	1.14	.008	4.56		ADHD = ADHD+ANX < ANX
SES	48.08	8.37	49.27	8.37	47.59	9.24	.77, *ns*		8.68	–
Maternal education level (P/HS/A/B/G)	2/7/12/15/23		1/2/4/12/9		0/1/3/8/11		.79, *ns*		6.69 x 10^3^	–
WISC-V SFIQ (Std. Score)	100.20	14.80	104.50	13.43	102.40	11.48	.40, *ns*		5.01	–
Anxiety Diagnoses (N/Y)										
Generalized AD	59/0		17/11		14/9		<.001	6.65 x 10^5^		ANX = ADHD+ANX > ADHD
Social AD	59/0		18/10		16/7		<.001	3.18 x 10^4^		ANX = ADHD+ANX > ADHD
Separation AD	59/0		26/2		22/1		.14, *ns*		15.16	ANX = ADHD+ANX = ADHD
Other Specified AD	59/0		22/6		15/8		<.001	2.64 x 10^3^		ANX = ADHD+ANX > ADHD
Specific Phobia	59/0		23/5		22/1		.003	2.52		ANX = ADHD+ANX > ADHD
ADHD Symptoms (T-scores)										
BASC-3 Attention Pxs										
Parent	68.75	7.10	69.43	7.82	64.73	8.48	.06, *ns*		1.22	–
Teacher	68.92	6.38	63.04	10.37	58.85	7.95	<.001	4.96 x 10^3^		ADHD > ADHD+ANX = ANX
BASC-3 Hyperactivity/Imp										
Parent	67.14	13.93	70.14	8.78	61.27	14.47	.05		1.11	ADHD = ADHD+ANX > ANX
Teacher	65.97	16.01	60.50	14.75	51.20	9.53	<.001	39.54		ADHD > ANX = ADHD+ANX
Working Memory Recall Performance (% Stimuli Correct)										
Word Span										
Congruent Trials	.53	.22	.53	.23	.77	.10	<.001	1.13 x 10^3^		ANX > ADHD = ADHD+ANX
Incongruent Trials	.52	.22	.51	.23	.75	.14	<.001	298.96		ANX > ADHD = ADHD+ANX
Stroop Span										
Congruent Trials	.53	.20	.59	.21	.72	.16	<.001	39.24		ANX > ADHD = ADHD+ANX
Incongruent Trials	.52	.22	.57	.23	.72	.17	<.001	33.91		ANX > ADHD = ADHD+ANX
Response Times (Color Naming) (RTs; milliseconds)										
Word										
Congruent Trials	1362.16	391.11	1429.61	463.01	1098.10	179.32	<.001	6.22		ANX < ADHD = ADHD+ANX
Incongruent Trials	1405.89	409.56	1524.37	509.63	1140.58	213.63	<.001	8.20		ANX < ADHD = ADHD+ANX
Stroop										
Congruent Trials	1349.75	406.18	1348.07	344.07	1242.16	294.92	.36, *ns*		5.92	–
Incongruent Trials	1665.84	554.05	1719.64	572.97	1484.03	397.13	.15, *ns*		3.73	–
Word Span										
Congruent Trials	1537.17	474.08	1729.43	521.62	1555.31	540.58	.26, *ns*		3.28	–
Incongruent Trials	1648.50	622.85	1704.68	467.19	1660.70	721.63	.89, *ns*		9.98	–
Stroop Span										
Congruent Trials	1628.65	611.84	1735.00	498.64	1532.50	562.27	.40, *ns*		5.83	–
Incongruent Trials	1928.15	666.43	2053.63	634.94	1814.63	501.77	.33, *ns*		5.26	–
Accuracy (Color Naming) (% Stimuli Correct)										
Word										
Congruent Trials	0.99	0.03	0.99	0.02	0.998	0.01	.06, *ns*		5.04	–
Incongruent Trials	0.98	0.04	0.99	0.02	0.997	0.01	.02		2.13	ANX > ADHD; ADHD = ADHD+ANX; ANX = ADHD+ANX
Stroop										
Congruent Trials	0.996	0.02	0.98	0.10	0.998	0.01	.34, *ns*		3.11	–
Incongruent Trials	0.97	0.04	0.96	0.10	0.97	0.03	.67, *ns*		6.34	–
Word Span										
Congruent Trials	0.98	0.04	0.97	0.08	0.999	0.01	.002		1.41	ANX > ADHD; ADHD = ADHD+ANX; ANX = ADHD+ANX
Incongruent Trials	0.97	0.07	0.96	0.09	0.995	0.01	.003		2.43	ANX > ADHD; ADHD = ADHD+ANX; ANX = ADHD+ANX
Stroop Span										
Congruent Trials	0.98	0.03	0.96	0.09	0.996	0.01	<.001	1.31		ANX > ADHD; ADHD = ADHD+ANX; ANX = ADHD+ANX
Incongruent Trials	0.95	0.07	0.95	0.06	0.98	0.03	.045		3.40	ANX > ADHD; ADHD = ADHD+ANX; ANX = ADHD+ANX

BF_10_, Bayes Factor for the alternative hypothesis over the null hypothesis. BF_01_, Bayes Factor for the null hypothesis over the alternative hypothesis (BF_01_=1/BF_10_). P-values are not corrected for family-wise error, and are included to allow interested readers to compare Bayesian and frequentist results. AD, Anxiety Disorder; BASC, Behavior Assessment System for Children. Ethnicity; B, Black or African American; H, Hispanic or Latino; M, Multiracial; White, White Not Hispanic; WISC-V, Wechsler Intelligence Scale for Children, Fifth Edition.Maternal education: P = partial high school, HS = high school diploma, A = associate’s degree, B = bachelor’s degree, G = graduate degree. ns = not significant.

### Manipulation check

Evidence supporting the success of the separate working memory and inhibitory control experimental manipulations would be (1) for the inhibitory control manipulation, evidence of significant decreases in response times and/or accuracy during incongruent relative to congruent trials only for the high inhibition tasks (indicating that the high inhibition conditions elicited significantly higher stroop interference effects than the low inhibition conditions), and (2) for the working memory manipulation, significantly lower correct recall rates during high working memory (encoding + recall) conditions relative to correct encoding rates during the low working memory (encoding-only) conditions (indicating that the high working memory conditions successfully required working memory processes). As detailed below, both experimental manipulations were successful (i.e., the data were >500 million times more likely under the hypothesis that the manipulations were successful than under the null hypothesis that they were unsuccessful).

Specifically, there was decisive evidence that our manipulation to increase inhibitory control demands successfully evoked high inhibition demands as evidenced by significant Inhibition Demands (Low, High) x Trial Type (Incongruent, Congruent) interactions for both response times (BF_10_=5.33 x 10^8^, p<.001) and accuracy (BF_10_=76.50, p=.002). *Post-hoc*s confirmed the success of the manipulation because this effect was specific to the high inhibition conditions, with the difference between incongruent and congruent trials significant for both RT and accuracy during the high inhibition (RT: d=-0.60, BF_10_=1.72 x 10^6^, p<.001; accuracy: d=0.38, BF_10_=2.77 x 10^3^, p<.001) but not low inhibition (RT: BF_01_=1.23, p=.07; accuracy: BF_01_=3.07, p=.18) conditions. In other words, interleaving incongruent and congruent trials within the stroop tasks successfully increased inhibitory control demands relative to the non-stroop tasks as intended.

Similarly, the evidence decisively supported an effect of working memory load on performance (Working Memory Low/High: BF_10 =_ 3.85x10^14^, p<.001), such that the high working memory conditions (encoding+recall) evoked higher working memory demands than the low working memory (encoding-only) conditions as intended. Given the success of these manipulations, we next examine whether each manipulation evoked performance decrements on tests/metrics intended to measure the other executive function, and whether these hypothesized effects differentially affected children with ADHD.

### Tier 1: effects of inhibitory control demands on working memory performance (working memory performance as DV)

**Figure 2:**
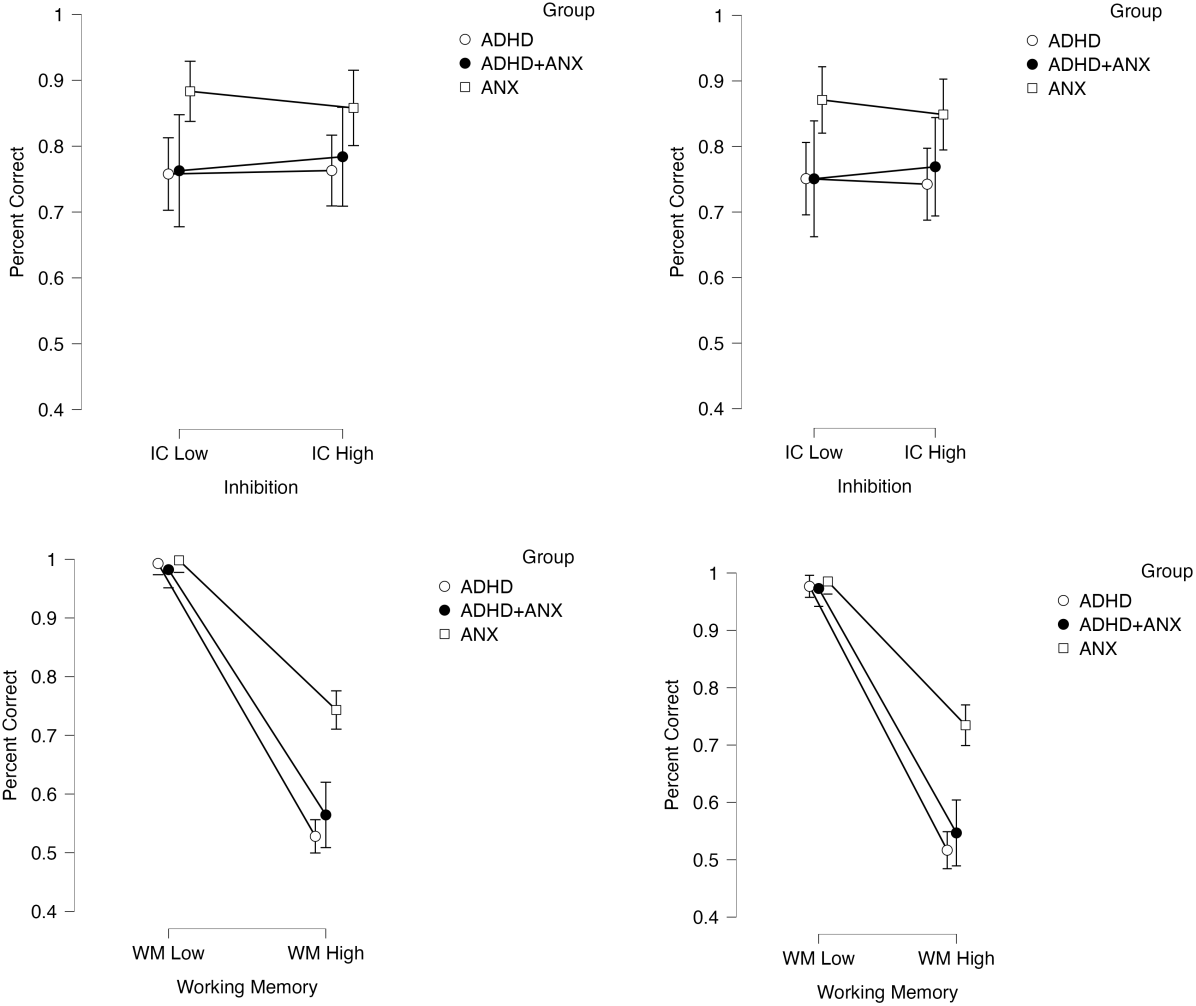
Effects of the inhibition and working memory manipulations on working memory performance (DV: percent correct) as a function of experimentally increasing inhibition demands (top row) and increasing working memory demands (bottom row). Effects are shown separately for congruent trials (left) and incongruent trials (right column). Error bars reflect 95% confidence intervals.

The 2 (within-subjects factor Inhibition Demands: low/high) x 2 (within-subjects factor Working Memory Demands: low/high) x 2 (within-subjects factor Trial Type: incongruent/congruent) x 3 (between-subjects factor Group: ADHD, ADHD+ANX, ANX) mixed-model ANOVA with working memory performance as the DV provided significant evidence for main effects of group (BF_10_=2.79 x 10^3^, p<.001; described below) and trial type (BF_10_=3.65, p=.008, d=0.09; slightly better recall for congruent stimuli). Please see the *Manipulation Check* section above for the main effect of the working memory factor on working memory performance (**Figure 2**, bottom row). There was strong evidence for the group x working memory interaction (BF_10_=2.30 x 10^3^, p<.001, *d*=1.00) with *post-hoc* tests indicating that all 3 groups showed lower encoding+recall during the high working memory conditions vs. their encoding during the low working memory conditions (*d*=1.69-3.09). The interaction was due to larger decreases in recall accuracy (% correct) for the ADHD and ADHD+ANX groups, relative to the ANX-only group. Specifically, the 3 groups did not differ statistically during the low working memory conditions (BF_01_=1.48-3.85, p>.99). In contrast, the ADHD (BF_10_=59.86, p<.001, d=1.45) and ADHD-ANX (BF_10_=100.15, p<.001, d=1.23) groups showed similar large magnitude impairments relative to the ANX group under high working memory conditions (ADHD/ADHD+ANX: BF_01_=2.76, p=.93; **Figure 2**, bottom row).

Importantly, there was strong evidence *against* the hypothesis that increasing inhibition demands would impact working memory performance (main effect of inhibition demands: BF_01_= 8.52, p=.85; inhibition x trial type interaction: BF_01_=6.25, p=.55) (**Figure 2**, top row). There was also evidence against the group x inhibition demands (BF_01_=2.51, p=.39), inhibition demands x working memory demands (BF_01_=10.75, p=.34), group x inhibition demands x trial type (BF_01_=10.42, p=.81), and the 4-way interaction (BF_01_=5.62, p=.66), indicating that experimentally increasing inhibition demands failed to impact working memory performance in clinically evaluated children with ADHD and/or anxiety. Taken together, these findings indicate that ADHD is associated with large magnitude impairments on working memory tests, while providing significant evidence *against* the hypothesis that these impairments are secondary to underlying inhibitory control deficits that affect working memory performance. In other words, inhibitory control processes do not appear to affect the performance of children with ADHD (with or without anxiety) on tests of working memory.

### Tier 2: effects of working memory demands on inhibitory control performance (response times and accuracy as DVs)

**Figure 3:**
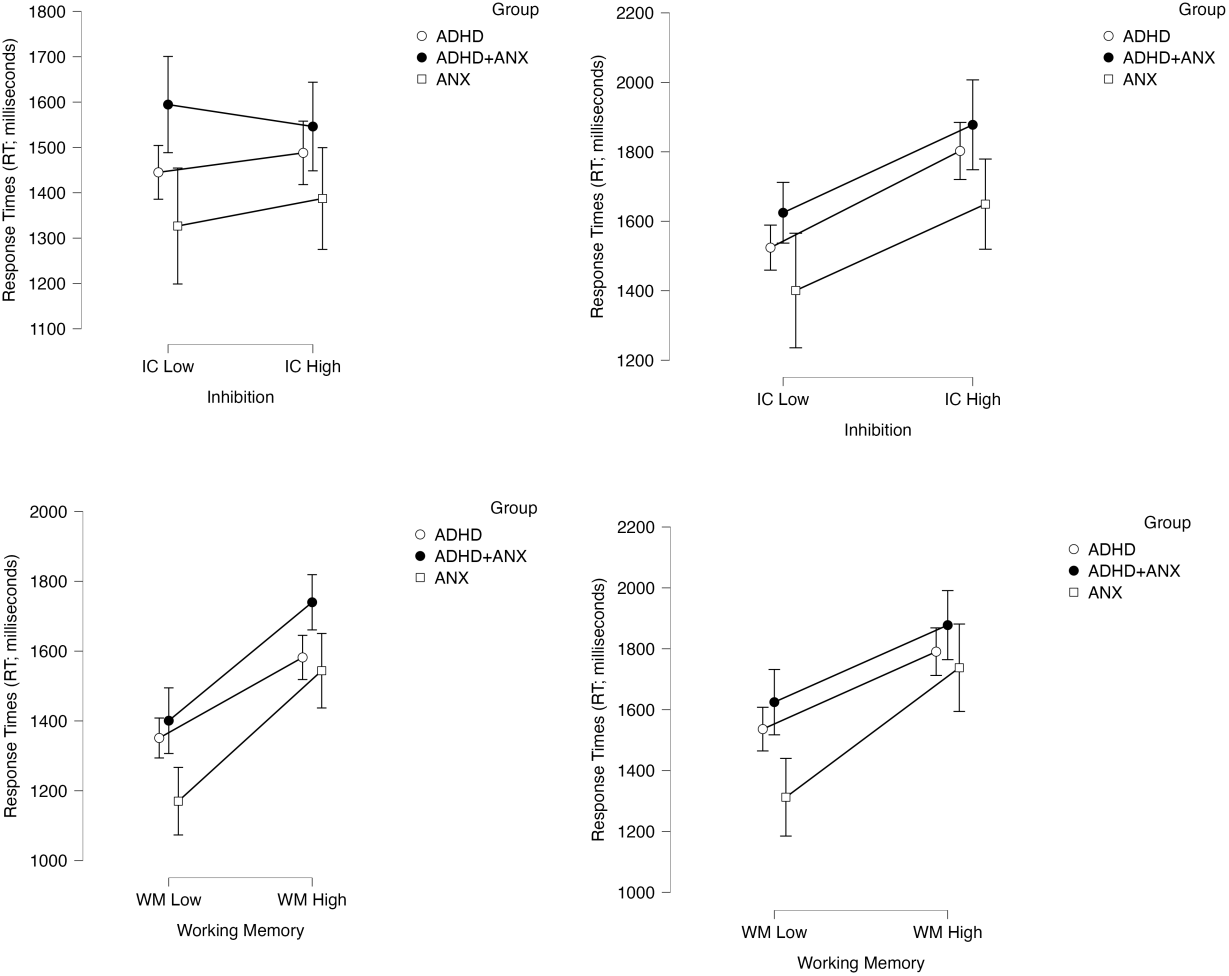
Effects of the inhibition and working memory manipulations on response times to correct color naming responses (DV: milliseconds) as a function of experimentally increasing inhibition demands (top row) and increasing working memory demands (bottom row). Effects are shown separately for congruent trials (left) and incongruent trials (right column). Error bars reflect 95% confidence intervals.

*Response time model.* Results of the 2 (within-subjects factor Inhibition Demands: low/high) x 2 (within-subjects factor Working Memory Demands: low/high) x 2 (within-subjects factor Trial Type: incongruent/congruent) x 3 (between-subjects factor Group: ADHD, ADHD+ANX, ANX) mixed-model ANOVA indicated decisive support for an effect of increasing working memory demands on slowing response times during the primary color identification tasks (main effect of working memory demands: BF_10_=3.71 x 10^9^, p<.001, d=1.53). Please see the *Manipulation Check* section above for the main effect of the inhibition manipulation on interference-related slowing (**Figure 3**, top row). In contrast, there was no evidence for, and in most cases significant evidence *against*, group x working memory demands (BF_01_=3.14, p=.24), working memory demands x inhibition demands (BF_01_=5.24, p=.98), group x working memory demands x trial type (BF_01_=3.64, p=.10), and the 4-way interaction (BF_01_=2.34, p=.62). These findings indicate that experimentally increasing working memory demands affects the inhibition and non-inhibition components of these tasks equivalently for clinically-evaluated children (**Figure 3**, bottom row). Notably, there was also no evidence for, and in most cases significant evidence *against*, effects of group (BF_01_=1.29, p=.17), group x inhibition demands (BF_01_=10.75, p=.82) and group x inhibition demands x trial type (BF_01_=5.38, p=.41), indicating that children with ADHD (with or without anxiety) did not demonstrate impaired inhibition (based on response speeds) relative to the ANX group.

**Figure 4:**
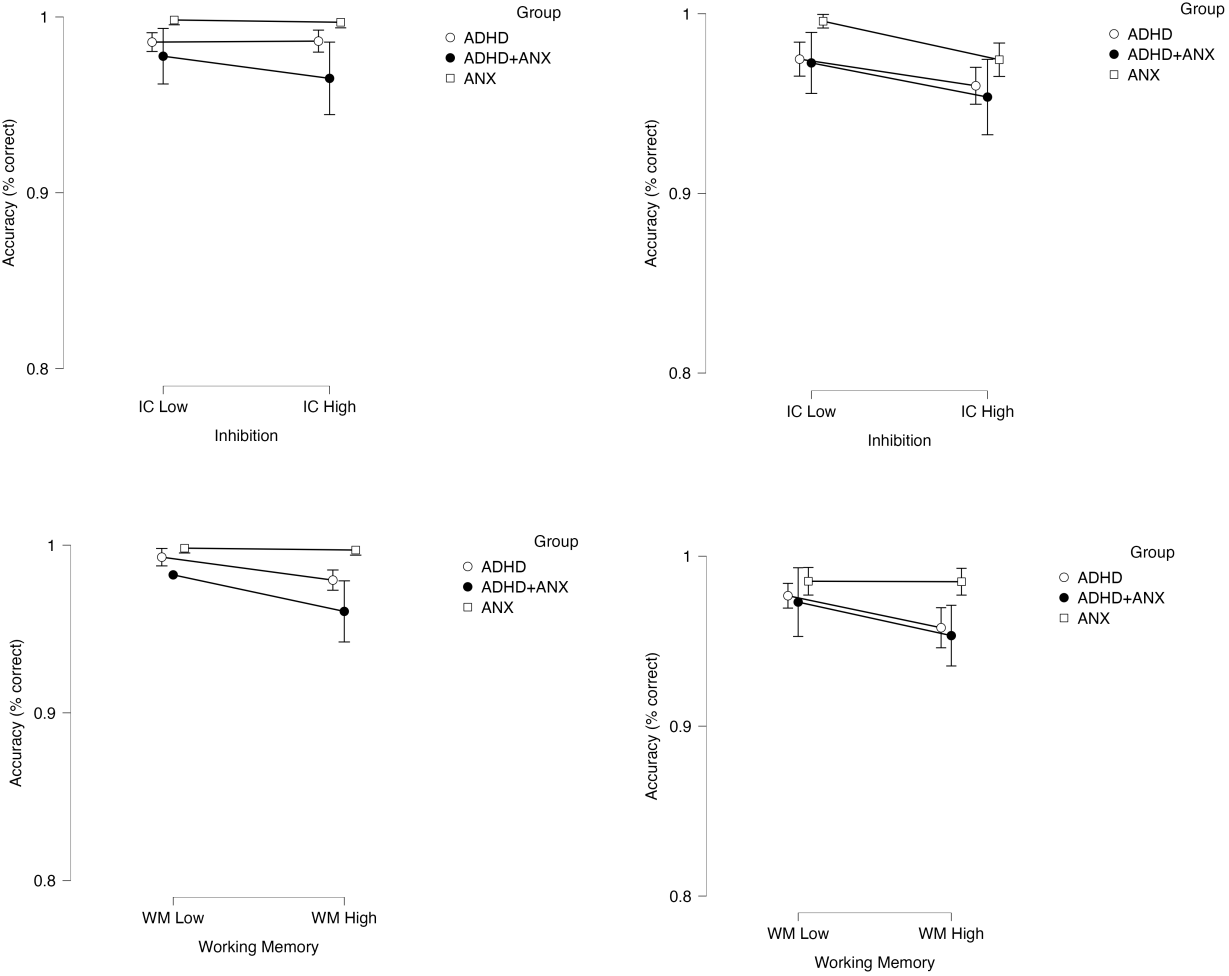
Effects of the inhibition and working memory manipulations on color naming accuracy (DV: percent correct) as a function of experimentally increasing inhibition demands (top row) and increasing working memory demands (bottom row). Effects are shown separately for congruent trials (left) and incongruent trials (right column). Error bars reflect 95% confidence intervals.

*Accuracy model.* Results of the 2 (within-subjects factor Inhibition Demands: low/high) x 2 (within-subjects factor Working Memory Demands: low/high) x 2 (within-subjects factor Trial Type: incongruent/congruent) x 3 (between-subjects factor Group: ADHD, ADHD+ANX, ANX) mixed-model ANOVA indicated decisive support for an effect of increasing working memory demands on reducing accuracy during the primary color identification tasks (main effect of working memory demands: BF_10_=317.42, p<.001, d=0.67). The main effect of the inhibition manipulation on interference-related accuracy reductions is described in the *Manipulation Check* section above (**Figure 4**, top row). There was significant evidence *against* the working memory demands x inhibition demands (BF_01_=9.35x10^5^, p=.74), group x working memory demands x trial type (BF_01_=10.75, p=.53), and the 4-way interaction (BF_01_=100.00, p=.64), indicating that experimentally increasing working memory demands equally affected children’s accuracy on both the inhibition and non-inhibition components of these tasks.

In contrast, there was a significant main effect of group (BF_10_=3.79, p=.05, d=0.47; ADHD = ADHD+ANX < ANX), and the group x working memory demands interaction was supported based on *p*-value but not Bayes Factor (BF_01_=1.46, p=.04, d=0.41).^5^ *Post-hoc*s for these effects indicated that both ADHD groups showed reduced accuracy across tasks relative to the ANX group. Specifically, the interaction was attributable to the ADHD group (BF_10_=734.4, p=.007, d=0.31) and potentially the ADHD+ANX group (BF_10_=1.88, p=.03, d=0.38) demonstrating significant reductions in color naming accuracy when working memory demands were increased, whereas this manipulation failed to affect accuracy for the ANX-only group (BF_01_=8.40, p>.99; **Figure 4**, bottom row).

Finally, there was significant evidence *against* effects of group x inhibition (BF_01 =_ 9.90, p=.53) and group x inhibition x trial type (BF_01_=7.30, p=.53). Combined with the group/group x working memory *post-hoc*s reported above, these findings indicated that the significant main effect of group was attributable to similarly reduced accuracy across the inhibition and non-inhibition components of these tasks, combined with potentially disproportionate reductions across the inhibition and non-inhibition task components as working memory demands increased for children with ADHD (with and without anxiety) relative to children with ANX.

Overall, results of the primary analyses (a) confirmed that our manipulations of working memory and inhibition were successful; and (b) demonstrated that experimentally occupying clinically evaluated children’s limited capacity working memory system produces slower response times and reduced accuracy on inhibition tasks – and does so equivalently across the inhibition and non-inhibition components of these tasks. For children with ADHD specifically, these results also (c) provided evidence *against* conceptual models that view working memory deficits as secondary outcomes of underlying inhibition deficits in ADHD; (d) indicated that children with ADHD with and without co-occurring anxiety exhibited similar, large magnitude working memory deficits (d=1.23-1.45); (e) showed that children with ADHD exhibit reduced accuracy on inhibition tasks (d=0.47), but that this impairment was not attributable to the tasks’ inhibition demands (i.e., the difficulties were equivalent across the low and high inhibition conditions); and (f) provided evidence that increasing working memory demands may differentially reduces accuracy (but not response times) on inhibition tests for children with ADHD, and also exerts this influence on the non-inhibition components of these tasks. Taken together, working memory appears to reflect an underlying mechanism that broadly affects children’s ability to inhibit prepotent tendencies and maintain fast and accurate performance more generally. Working memory may also explain, in large part, the impairments that children with ADHD exhibit on accuracy-based estimates of inhibitory control. Conversely, ADHD-related inhibition deficits, when present, do not appear to be responsible for ADHD-related difficulties on working memory complex span tests. More generally, depleting inhibitory resources via a stroop paradigm did not interfere with children’s working memory performance.

### Tier 3: sensitivity analyses

Finally, we conducted a series of sensitivity analyses to probe the robustness of our findings and impact of our *a priori* decisions to (a) exclude age as a covariate to conserve power; (b) include children with co-occurring ASD in the sample; (c) retain children diagnosed with reading disabilities in the sample; and (d) categorize children whose only anxiety diagnosis was specific phobia as ANX/ADHD+ANX. Reporting is truncated for readability. First, we repeated the primary Tiers 1 and 2 models, this time covarying child age given the unexpected finding that the ANX group was slightly older than both ADHD groups. Age did not produce a significant main effect or interact with any main or interaction terms in the models with working memory performance (p>.08, BF_01_>1.47) or inhibition accuracy (p>.29, BF_01_>3.58). In contrast, in the inhibition RT (speed) model, age demonstrated a significant main effect (p<.001, BF_10_=1.42x10^3^; older children demonstrated faster response times) but age did not interact with any main or interaction terms in the models to affect response times (p>.08, BF_01_>1.43).

Next, we repeated the primary analyses a second time, this time excluding children with ASD (*n*=15). Results of all models were unchanged with these children excluded, with one minor exception: The main effect of group in the inhibition accuracy model became non-significant at p=.06 (BF_10_=1.08) despite a near identical effect size compared to the primary analyses (d=0.50 with ASD excluded vs. 0.48 in the primary model), suggesting this was likely an artifact of lower power rather than specific to our decision to retain children diagnosed with ASD in our study. We then tested the extent to which the results were impacted by our retention of children with reading disabilities in the sample given that reading the color words is necessary to evoke their interference effects. As noted above, all children were able to fluently read the color words based on practice trials, and the manipulation check provided decisive evidence that the high inhibition tasks elicited the expected interference effects. Thus, it was unsurprising that the pattern and interpretation of results was unchanged when children with reading disabilities (n=17) were excluded. Finally, we probed our decision to categorize children with specific phobias (all 6 = phobias of the dark) with the other anxiety disorders, given that the novel, evaluative setting with unfamiliar adults likely did not evoke anxiety symptoms that might interfere with test performance for these children like it presumably would for children with anxiety disorders characterized by performance evaluation (social, generalized) and/or separation worries (81). The pattern and interpretation of results was unchanged with these children excluded.

## Discussion

The current study was among the first to use a dual-dissociation experimental design to systematically manipulate demands on both working memory and inhibitory control in a relatively large, clinically evaluated, and carefully phenotyped sample of children with ADHD and/or anxiety, with implications for conceptual models of the primacy and relevance of core executive functions in ADHD. Confidence in the findings is supported by study strengths including (a) decisive evidence that both experimental manipulations were successful, (b) comparison of the ADHD group to a clinical comparison group of children with anxiety disorders as well as a group of children with both ADHD and anxiety, (c) relatively large sample size (for clinical child research), (d) adoption of the experimental stroop paradigm that provides improved construct validity relative to classic neuropsychological versions (99), and (e) the experimental design that allows stronger conclusions regarding causality.

Overall, we found strong evidence that depleting inhibitory resources (via a stroop interference paradigm) does *not* impact children’s performance on working memory tests – a null finding that was equivalent for all three clinical groups. In contrast, we found decisive evidence that occupying children’s limited capacity working memory system (by adding complex span-style recall demands) affects both speed and accuracy on inhibition tests, and appears to differentially affect the ability of children with ADHD (with and without anxiety) to maintain high levels of accuracy on inhibition tasks. Interestingly, working memory broadly impacted children’s performance, in that it impacted both the inhibition and non-inhibition components of the inhibition tests equally. Expanded discussion of these findings and implications for theoretical models of ADHD are discussed below.

### (Null) effects of inhibitory control processes on working memory performance

As noted above, experimentally increasing inhibition demands failed to affect children’s performance on working memory tests, with Bayesian statistics providing strong/decisive support for the null hypothesis that inhibition (a) is *not* a causal factor affecting performance on working memory tests for clinically-evaluated children and (b) cannot explain the working memory difficulties exhibited by children with ADHD. Thus, these findings directly contradict conceptual models predicting that inhibition deficits underlie working memory difficulties in children with ADHD. Instead, this pattern of results is consistent with recent clinical trial data indicating that targeted training of inhibitory control does not produce downstream improvements in working memory for children with ADHD (29). It is consistent also with evidence that experimentally increasing inhibition demands does not disrupt computationally modeled cognitive information processing or encoding/motor processes, but rather equivalently causes children with and without ADHD to adopt more cautious response styles (63).

In contrast, at first glance these findings appear to contradict the only other (to our knowledge) experiment to manipulate both working memory and inhibitory control in ADHD, which found that depleting inhibition resources using a stop-signal paradigm produced significant reductions in *n*-back accuracy for children with and without ADHD (30). At the same time, *n*-back tasks have been criticized as measures of working memory because they correlate poorly with complex span tests of working memory (meta-analytic *r*=.20; 110), likely because *n*-back tasks require only passive recognition. In contrast, complex span tasks require active recall processes (111), recruit different cortical regions (112), and produce performance differences under otherwise identical conditions (14, 113).

Notably, the current study evoked inhibition demands via a stroop paradigm that is thought to evoke both response inhibition and interference control subcomponents of the inhibitory control construct, whereas Alderson et al. (30) used a stop-signal paradigm that is typically considered an index of response inhibition. Thus, it may be that only specific subcomponents of inhibitory control influence working memory processing. This conclusion appears unlikely, however, based on experimental evidence indicating that both task types share a common inhibitory control mechanism (98, 114) and factor analytic evidence indicating that both tasks load together (e.g., 5, 17). Instead, the discrepancy between the current study and Alderson et al. (30) might be best understood through the lens of the Fosco et al. (63) experiment described above. In this view, adopting a more cautious response strategy when inhibition demands are increased reduces the likelihood that children will be able to recognize and respond quickly enough when a letter repeats itself (i.e., respond correctly on an *n*-back task), whereas inhibition’s lack of impact on cognitive information processing would be less likely to affect the more cognitively demanding task of retaining information in working memory in the face of interference (29).

### Effects of working memory processes on inhibitory control performance

There was decisive evidence that working memory is important for fast and accurate responding on inhibition tasks. These findings appear to support conceptual models predicting that working memory deficits underlie difficulties on inhibition tasks for children with ADHD. However, a more nuanced interpretation appears warranted based on careful inspection of the results. First, increased working memory demands appear to disproportionately affect accuracy for children with ADHD, with equivalent effects on the inhibition and non-inhibition components of the tasks, indicating that failure to account for working memory is likely to result in overestimates of inhibition deficits in ADHD by approximately *d*=0.41-0.50 when using accuracy-based scores. In contrast, working memory also impacts response speeds on inhibition tests, but appears to have similar impact across the three clinical groups. Perhaps more importantly, the robust impact of working memory occurred across both low and high inhibition conditions and across congruent and incongruent trials – regardless of whether accuracy or response times were used to estimate performance. This pattern indicates that working memory broadly affects children’s performance on inhibitory and non-inhibitory components of these types of tasks. This pattern of results was consistent with prior experimental evidence demonstrating that occupying children’s limited capacity working memory system disrupts computationally modeled processing speed for children with and without ADHD (85). It is also broadly consistent with a recent RCT indicating that training working memory may produce general improvements on both inhibition and choice-response tasks for children with ADHD (29, 115). In contrast, increasing memory demands using an *n*-back paradigm failed to affect inhibition performance in Alderson et al. (30) – through the lack of effect in that study was likely because the high memory condition was too difficult for both groups as noted above.

### Implications for ADHD neurocognitive research

Taken together, these findings have several implications for neurocognitive research in ADHD. First, the dual dissociation finding that working memory affected inhibition task performance but not vice versa argues against the simple view that doing two tasks at once is always more difficult than doing one task. It is also inconsistent with models suggesting a non-specific effect in which engaging any executive function process produces generalized reductions in subsequent performance on executive functioning more broadly (75). Instead, the current and prior findings (e.g., 29) indicate that, for clinically evaluated children with and without ADHD at least, it matters what those tasks/processes are, and how they are combined. Second, the finding that working memory is a directional, if not causal, mechanism underlying performance on both the inhibition-specific and non-inhibition aspects of inhibition tests urges caution when interpreting the results of inhibition tests for children with ADHD, and clinically evaluated children more generally. In particular, the current results suggest that neuropsychological and research tests of inhibitory control that rely on a single score (e.g., accuracy, commission errors, response times) are likely to be particularly confounded by the tests’ working memory demands. As such, inhibition scores that control for performance on the non-inhibition components of the test are likely to provide more construct valid estimates of inhibitory processing specifically – particularly when used as part of a battery of inhibition tests that can be combined statistically to produce latent performance estimates (e.g., 99).

Third, the finding that working memory difficulties appear to exaggerate estimates of inhibition deficits in ADHD by *d*=0.41-0.50 (for accuracy) is striking given that it falls squarely within the range of meta-analytic estimates of inhibitory control deficits in ADHD (*d*=-0.03 to 0.63; 24, 26–28). Although these effect sizes are not directly comparable because they reflect performance changes vs. between group differences, this finding is consistent with evidence that only 5-11% of children with ADHD have inhibition deficits without co-occurring working memory deficits (9, 74) and calls into question the extent to which children with ADHD have deficits in inhibitory control versus perform poorly on inhibition tests due to their underlying working memory difficulties (45).

Taken together, the current findings appear most consistent with conceptual models that place working memory as an underlying causal mechanism affecting performance on inhibitory control tasks (e.g., 14), with the caveat that there appears to be a small subset of children with ADHD who have inhibition difficulties that cannot be explained by working memory difficulties as noted above. In this view, environmental demands that challenge working memory (in this case by adding a concurrent memory load) interact with a preexisting neurobiological vulnerability (e.g., underdeveloped cortical structures that support working memory; 44) to produce secondary impairments including goal maintenance failures (61), reduced information processing efficiency (85), and reduced attentional filtering (65). In turn, these secondary impairments result in failure to inhibit when needed as well as more general lapses of attention (e.g., 116) that broadly reduce accuracy. In contrast, working memory appears to affect response speeds more similarly for children with ADHD and/or anxiety, which is broadly consistent with prior experimental and RCT findings (55, 85, 115).

Finally, this pattern of results is consistent with recent calls to reconceptualize inhibition as an outcome rather than a process/mechanism that produces outcomes (117). In this view, inhibition is not something we *use* to suppress a response; instead, the *goal* is to inhibit and we rely on other processes to do so successfully (117), including engaging working memory (this study) to adopt more cautious response strategies (63), maintain task goals (61, 64), and filter out irrelevant information (65).

### Limitations

The following caveats should be considered. First, due to funding constraints we were unable to recruit a typically developing control group. Although both ADHD groups differentiated themselves from the anxiety disorders comparison group, and the anxiety group performed similarly to the non-ADHD groups in our prior experiments (with non-overlapping samples; e.g., 63, 85), anxiety may be associated with small impairments, or potentially a small strength, across executive functions and thus the obtained effect sizes may be modest over- or under-estimates of ADHD-related impairments more broadly. Replications that include a typically developing group are warranted. Similarly, the strength of support for the differential impact of working memory on inhibition accuracy for children with ADHD vs. ANX (*d*=0.50) was only twice as likely under the alternative vs. null hypothesis (i.e., BF_01 =_ 2.03), requiring more tentative conclusions. Second, we used a complex span-based verbal working memory task and a stroop-interference inhibition task. Replications that systematically manipulate additional working memory processes (e.g., continuous updating, serial/temporal reordering; 63), additional short-term storage subsystems (e.g., spatial storage/rehearsal; 22), as well as additional exemplars/subcomponents of inhibitory control (e.g., action restraint/cancellation; 45) are needed despite their consistency with prior work in the cognitive literature (e.g., 61). Finally, although our experimental manipulations were successful in evoking their target mechanisms, they may have also evoked increases in other processes as well. Experimental studies of those mechanisms/processes are needed to understand the extent to which the reported effects were specifically attributable to working memory/inhibition.

## Conclusions and future directions

Overall, the current findings are consistent with evidence from the cognitive literature and prior ADHD experimental work implicating working memory capacity as a core, underlying mechanism that broadly affects performance across a variety of neurocognitive tasks (e.g., 85, 115). The findings also highlight the importance of differentiating between neurocognitive abilities and neurocognitive test performance. A significant proportion (if not the majority) of the variance in any neuropsychological/neurocognitive test is attributable to factors other than the construct(s) of interest (i.e., the ‘*task impurity problem*’; 99); thus, the use of multiple tests per construct and control for known processes that impact performance on tests of the constructs of interest is warranted (e.g., accounting for working memory when studying inhibitory control as suggested by the current findings). Future work is also needed to identify ‘mechanisms of the mechanisms’ (e.g., potential factors beyond working memory that affect working memory test performance).

More broadly, the field would benefit from increased application of the experimental psychopathology framework to determine the impact of these executive functions, and other putative causal mechanisms, in producing ADHD behavioral symptoms and functional impairments (e.g., 63, 116, 118). Experimental methodologies hold considerable promise for complementing longitudinal findings and helping to differentiate among competing conceptual models of ADHD. For example, most longitudinal studies have linked improvements in working memory, or executive functioning more generally, with remission of ADHD symptoms (e.g., 74). Interestingly, however, these findings are equally supportive of (1) models that position working memory/executive functioning as underlying causes of ADHD – i.e., when the underlying impairments/causes become less severe, so do the behavioral outcomes/effects of those impairments, and (2) models that view working memory/executive functioning as non-causal factors that instead help compensate for persisting impairments in other domains. In both cases, the models predict executive/behavioral associations over time. In contrast, experimental studies can provide clear evidence for/against these competing models because only the causal models predict that environmental demands that challenge these children’s underdeveloped executive function(s) will produce measurable, in-the-moment increases in ADHD behaviors (e.g., 73, 118). Nonetheless, experimental studies are unable to document potential cumulative effects of neurocognitive difficulties over time, track development across the lifespan, or determine how growth in executive functioning affects ADHD symptom presence/severity. Longitudinal studies are also clearly needed. In contrast, conclusions from ADHD cognitive training studies have been highly limited because most protocols have not shown large enough improvements in the trained/targeted cognitive abilities to realistically expect detectable downstream behavior changes, even if the causal models are correct (for review see 119) – although newer neurocognitive training protocols appear to be showing more robust improvements in their target mechanisms (e.g., working memory; 55, 120) and thus may hold promise for further clarifying the extent to which associations between executive function(s) and ADHD behaviors are causal vs. correlational.

## Data availability statement

The datasets presented in this study can be found in online repositories. The names of the repository/repositories and accession number(s) can be found below: Center for Open Science: https://osf.io/gts6x/.

## Ethics statement

The studies involving humans were approved by Florida State University Human Subjects Committee (STUDY00001032). The studies were conducted in accordance with the local legislation and institutional requirements. Written informed consent for participation in this study was provided by the participants’ legal guardians/next of kin.

## Author contributions

MK: Conceptualization, Formal analysis, Funding acquisition, Methodology, Supervision, Writing – original draft. NG: Conceptualization, Writing – review & editing. EC: Conceptualization, Writing – review & editing. CM: Writing – review & editing. AC: Writing – review & editing. FG: Writing – review & editing. EC: Writing – review & editing. MT: Writing – review & editing. LS: Conceptualization, Data curation, Writing – review & editing.

## Footnotes

1 Kofler MJ, Soto EF, Singh LJ, Harmon SL, Jaisle E, Smith JN, et al. Executive function deficits in attention-deficit/hyperactivity disorder and autism spectrum disorder. *Nat Rev Psychol*. (in press).

2 These percentages were not reported directly in Karalunas et al. (74), but were computed using the reported class overlap percentage, the reported number of children with ADHD in each class, and the total ADHD sample size.

3 As noted below, the pattern and interpretation of results was unchanged in sensitivity analyses excluding children whose only anxiety diagnosis was a specific phobia.

4 As recommended in the K-SADS, oppositional-defiant disorder (ODD) was diagnosed only with evidence of multi-informant/multi-setting symptoms.

5 We conducted exploratory analyses to test the hypothesis that the inconsistent frequentist/Bayes results for this model were due to excessive variability in the ADHD+ANX group relative to the ADHD and ANX groups (see 95%CIs in **Figure 4**). This hypothesis was confirmed: With the ADHD+ANX removed, the frequentist and Bayesian group x working memory interaction results for accuracy was more consistent (BF_10_=2.03, p=.02, d=0.50). Still, the strength of the evidence was less strong relative to the other significant results (i.e., the data were only twice as likely under the alternative hypothesis that an effect exists than under the null hypothesis of no effect). Thus, we discuss these findings using more tentative language (e.g., ‘potentially’, ‘appears to’). Significance/interpretation of all other main/interaction effects were consistent with the main text. For completeness, we also checked the RT and working memory recall models; the significance/interpretation of all main/interaction effects remained unchanged with the ADHD+ANX group removed.
